# Cerebral oxygenation responses during kangaroo care in low birth weight infants

**DOI:** 10.1186/1471-2431-8-51

**Published:** 2008-11-07

**Authors:** Esmot Ara Begum, Motoki Bonno, Noriko Ohtani, Shigeko Yamashita, Shigeki Tanaka, Hatsumi Yamamoto, Masatoshi Kawai, Yoshihiro Komada

**Affiliations:** 1 Clinical Research Institute and Department of Pediatrics, National Hospital Organization, Miechuo Medical Center, 2158-5 Hisaimyojin Cho, Tsu, Mie, 514-1101, Japan; 2 Department of Nursing, National Hospital Organization, Miechuo Medical Center, 2158-5 Hisaimyojin Cho, Tsu, Mie, 514-1101, Japan; 3 Masatoshi Kawai, PhD, Department of Developmental Clinical Psychological Institute for Education, Mukogawa Women's University, 6-46 Ikebiraki Cho, Nishinomiya, Hyogo, 633-8558, Japan; 4 Department of Pediatrics and Developmental Science, Mie University Graduate School of Medicine, 174-2 Edobashi, Tsu, Mie, 514-8507, Japan

## Abstract

**Background:**

Kangaroo care (KC) has been widely using to improve the care of low birth weight infants. However, very little is known about cerebral hemodynamics responses in low birth weight infants during KC intervention. The objective of this study was to elucidate the response of cerebral hemodynamics during KC in low birth weight infants.

**Methods:**

Near infrared spectroscopy measured regional cerebral oxygenation (rSO_2_), heart rate (HR), respiration rate (RR) measured by electrocardiogram, and percentages of oxygen saturation (SpO_2_) measured by pulse oxymetry was monitored in 16 preterm infants (< 1600 g) in three sessions: before, during, and after KC. Using power spectral analysis, total power (TP), low-frequency (LF, 0.02–0.20 Hz) and high-frequency (HF, 0.20–0.50 Hz) bands, the ratio of LF/HF were calculated and normalized as %LF or %HF = LF or HF/TP × 100 (%).

**Results:**

Significant differences were not observed in the mean rSO_2_, HR, and SpO_2 _throughout sessions; however, the TP of these parameters was significantly decreased during KC and increased after KC (p < 0.001). The %LF of LrSO_2 _and RrSO_2 _was decreased during KC (p < 0.05) with decreased %HF in RrSO_2 _(p < 0.05). The %LF of HR was significantly increased during KC while %HF was decreased (p < 0.05). Mean and TP of RR was increased during KC (p < 0.01 respectively) with the increase of quiet sleep state (p < 0.05) and decreased after KC (p < 0.01). The %LF of RR was increased after KC (p < 0.05) with decreased %HF (p < 0.05); however, significant changes were not observed during KC.

**Conclusion:**

KC intervention appears to have influence on cerebral hemodynamics as well as cardiorespiratory parameters. The results of rSO_2 _and HR might be associated with quiet sleep states. The results of this study may indicate the contribution of KC intervention to the activation of central nervous system and brain function. Further study is needed to determine the underlying physiology responsible for these differences.

## Introduction

Recently, skin – to – skin care, called kangaroo care (KC), has been widely practiced for preterm and low birth weight (LBW) infants in the neonatal intensive care unit (NICU). During kangaroo care, the mother holds a naked infant in a vertical position against the breasts so that the infant can achieve skin – to – skin contact. KC was first introduced in Bogotá, Columbia by Dr. Edgar Rey and Hector Martinez in 1978 as a way of compensation for the overcrowding of incubators in hospitals caring for preterm infants [1]. According to their report on KC, they have found improved outcomes in survival rates and health status. Now, KC is practiced not only in developing countries, but also in developed countries.

Many studies have been performed to evaluate the psychological and physiological responses during KC in preterm infants [2-4]. Positive psychological effects on mothers and mother – infant bonding are well recognized [5]; however, the physiological effects of KC are still inconclusive. Previously, it has been reported that KC improves thermal regulation [6-8], respiratory pattern and oxygen saturation [9,10], reduces apnea and bradycardia [9,11], accelerates weight gain [12], increases vagal tone responses [13], reduces activity level, and enhances the duration of quiet sleep [14,15] in preterm infants. On the other hand, increases in body temperature have been found to be associated with an increased frequency of apnea and bradycardia [16] and an increased oxygen requirement during KC was found in intubated infants [17]. Most of these studies were performed on cardiorespiratory parameters rather than cerebral hemodynamics. Preterm infants are highly susceptible to develop various cerebral lesions like intraventricular hemorrhage or periventricular leucomalacia following cerebral hypoperfusion because of their immature brain [18]. Cerebral oxygenation is one of the important parameters in cerebral hemodynamics, has been widely used to monitor cerebral perfusion in infants with birth asphyxia or brain lesions like hypoxic-ischemic encephalopathy [19-21]. The response of cerebral hemodynamics in accordance with sleep states was reported previously [22]; however, no study has yet been performed on the response of cerebral hemodynamics in preterm infants during KC intervention.

Spectral analysis of time series data using Fast Fourier transformation (FFT) has been widely utilized to study the autonomic nervous system [23-26]. In power spectral analysis, low – frequency region from 0.02 – 0.20 Hz is reflected sympathetic activities and high-frequency region from 0.2 – 2.0 Hz is reflected parasympathetic activities and total power, a index of total variance (the total area under the curve of power spectral density) and the ratio of LF/HF power reflects the balance between sympathetic and parasympathetic activities. Two studies have been reported on the heart rate variability (HRV) during KC using power spectral analysis [27,28]; however, their results were not conclusive. Further, previous studies on cerebral hemodynamics using power spectral analysis have reported the position dependent responses in adult [29], however, no study have been performed on the spectral characteristics of cerebral hemodynamics during KC position in preterm infants.

In this study, we investigated cerebral hemodynamics in addition to cardiorespiratory parameters in preterm infants during KC intervention using power spectral analysis. Therefore, we investigated regional cerebral oxygenation (rSO_2_) as a parameter of cerebral hemodynamics, heart rate (HR), respiratory rate (RR), and SpO_2 _in stable low birth weight infants during KC using power spectral analysis.

## Materials and methods

### Subjects

Nineteen preterm infants with birth body weight < 1,600 g and gestational age < 33 weeks (wks) were enrolled in this study. All of the infants were stable and breathed spontaneously without supplemental oxygenation with postconceptional age ≥ 32 wks. Infants who had severe congenital malformations, severe asphyxia, and a potential cause of apnea other than immaturity, such as sepsis or intracranial hemorrhage, were excluded from the study. Three infants were excluded because of interrupted observation. Finally, 16 infants were selected for further analysis (Table 1). The median gestational age was 28 wks (range, 24 – 33 wks) and median birth body weight was 1,228 g (range, 692 – 1,586 g). Postconceptional age on the day of the study was 36 wks (range, 33 – 42 wks) and body weight was 1,458 g (range, 946 – 1,858 g). Eight infants were born via caesarean section. Seven infants had received mechanical ventilation after birth for a median duration of 6 days. Four infants had received theophylline for apnea on the day of KC intervention.

**Table 1:** Characteristics of 16 low birth weight infants participating in Kangaroo care

**Subject number**	**Sex**	**GA (wks)**	**BBW (g)**	**Apgar score 1 min/5 min**	**Acute phase illness**	**At the day of Kangaroo care**		
						
						**PCA (wks)**	**BW (g)**	**Theophylline**
1	M	28	1228	8/8	PPHN	34	1531	Yes
2	M	28	1258	8/9	TTN	33	1458	Yes
3	F	31	1282	7/9	RDS	34	1222	No
4	F	30	1538	7/8	RDS	34	1482	No
5	F	30	1228	7/8	RDS	36	1298	No
6	F	28	1140	6/8	TTN	40	1638	No
7	F	27	1152	1/6	RDS	36	1298	No
8	M	33	1586	7/9	TTN	36	1752	No
9	M	24	692	8/8	RDS	40	1064	Yes
10	M	25	756	5/7	RDS	35	1043	Yes
11	M	27	1272	2/3	RDS	37	1581	No
12	M	25	866	5/8	RDS	41	1858	No
13	F	29	1106	9/9	RDS	36	1359	No
14	M	26	982	9/10	RDS	36	946	No
15	M	25	772	6/6	RDS	42	1334	No
16	M	27	1130	7/9	CLD	36	1540	No

GA: gestational age, BBW: birth body weight, PCA: postconceptional age, BW: body weight, PPHN: persistent pulmonary hypertension of the newborn, RDS: respiratory distress syndrome, TTN: transient tachypnea of the newborn

### KC and data collection

KC intervention was performed for one hour. The temperature of the room ranged from 27 to 28°C and the humidity ranged from 60 to 70% during KC. Infants were observed carefully and monitored in three conditions: 30 minutes in the incubators (before KC), 1 hour of KC intervention (during KC), and 30 minutes in the incubator again after KC intervention. Mothers were seated on a reclining chair at a 60° angle, wearing a front opening blouse. The infants were placed naked except for a diaper directly onto the skin between the breasts and covered with a light blanket. Infants were fed 1 hour before KC. All infants were continuously monitored with electrocardiogram to determine HR, RR and with pulse oxymetry for percentages of oxygen saturation (SpO_2_). Regional cerebral oxygenation was measured with a near infrared spectroscopy, NIRS (INVOS 4100, Somanetics, Troy, MI), with the two probes positioned on the bilateral frontoparietal areas. Physiological data were recorded at 10 second intervals (averaged over 10 second period) through a Wave Archiving System (WAS-J: Agilent Technologies, Inc.) and further analyzed. Data from the first 30 minutes during KC were excluded from the analysis to minimize the effects of changes due to the adaptation process. The data for rSO_2 _were recorded from both the left and right hemispheres independently (LrSO_2 _and RrSO_2_, respectively).

Behavioral states of infants were recorded throughout the observation period using the Brazelton Neonatal Behavior Assessment Scale [30]. Behavioral states of infant were observed by nurses trained on the observational scale. Five different behavioral states were observed: 1) Quiet sleep; 2) Active sleep; 3) Drowsiness; 4) Alert inactivity; and 5) Active awake. Behavioral states were judged before KC, during KC, at the end of KC, and 30 minutes after KC. Body temperature was measured at the beginning of data recording before the KC session and after the end of KC. Informed consents were obtained from the parents and the study was approved by the ethical committee of the institute.

### Power spectral analysis

**Figure 1:**
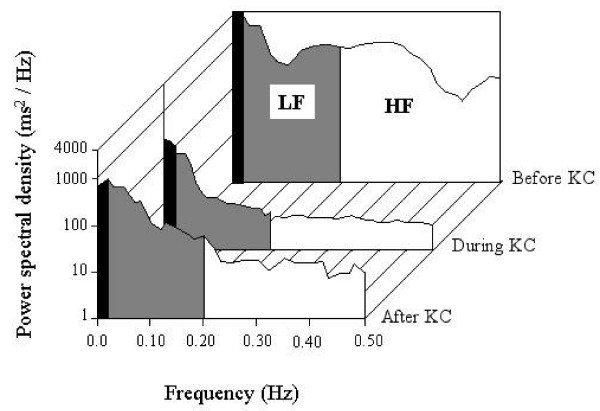
A typical graph of power spectral density (ms^2^/Hz) of right rSO_2 _displaying the power of the low frequency (LF: 0.02 – 0.2 Hz) and high frequency (HF: 0.2 – 0.5 Hz) bands before, during, and after the KC session.

Power spectral analysis was performed on rSO_2_, HR, RR, and SpO_2 _in three sessions as previously reported [28]. The power spectral density was calculated and divided into two frequency bands in each session as shown in Figure 1. The regional power of low – frequency (LF, 0.02 – 0.2 Hz) and high – frequency (HF, 0.20 – 0.5 Hz) bands, and the ratio of LF/HF were calculated. The powers of LF and HF were normalized using the formulas % LF = LF/TP × 100 (%) and % HF = HF/TP × 100 (%) [31]. Total power (TP) was obtained by integrating the power spectrum from frequency 0.02 to 0.50 Hz.

### Statistical analysis

Data were analyzed with SPSS 13.0 for Windows (SPSS Inc., Chicago, IL). A repeated measures ANOVA was performed to analyze the differences of parameters among three sessions. Conventional statistics: mean, standard deviation, coefficient of variance (CV), range, normal distribution, and median were performed for all parameters. Data are expressed as median (range) or mean ± SD.

## Results

### Descriptive statistical analysis

Descriptive statistics of HR, RR, SpO_2_, LrSO_2 _and RrSO_2 _are shown in Table 2. In mean HR, SpO_2_, LrSO_2_, and RrSO_2_, there were no significant differences among the three sessions; however, RR was significantly increased during KC and decreased after KC (p < 0.05, respectively). Body temperature was increased by 0.3°C during KC (p < 0.01). The CV of HR was decreased by 2.2% during KC (p < 0.05), while it was increased again after KC by 2.9% (p < 0.05). There were no significant differences in the CVs of RR, SpO_2_, LrSO_2_, and RrSO_2_.

**Table 2:** Central tendencies and coefficient of variances of physiological variables before, during and after KC intervention

**Variables**	**Measures**	**Before KC**	**During KC**	**After KC**
*Heart rate (bpm)*	Mean	149.4 ± 6.3	150.0 ± 10.0	150.4 ± 8.7
	CV	6.8 ± 3.2	4.6 ± 1.3*	7.5 ± 4.7 †
*Respiratory rate (bpm)*	Mean	39.4 ± 8.9	44.0 ± 5.1**	39.7 ± 7.9 † †
	CV	24.1 ± 5.4	22 ± 6.7	19.7 ± 7.6
*SpO_2_(%)*	Mean	98.0 ± 1.3	97.6 ± 2.5	97.5 ± 1.7
	CV	2.3 ± 1.4	1.5 ± 0.9	2.9 ± 2.4
*Left-rSO_2_(%)*	Mean	46.8 ± 5.6	47.3 ± 6.1	47.5 ± 7.3
	CV	6.6 ± 4.1	5.8 ± 3.3	4.1 ± 1.6
*Right-rSO_2_(%)*	Mean	48.6 ± 6.9	49.1 ± 9.4	47.8 ± 6.9
	CV	7.2 ± 4.6	7.2 ± 5.5	5.6 ± 2.3
*Body temperature, (°C)*	Mean	37.0 ± 0.2***	37.3 ± 0.3	
	Median (range)	37.1 (36.6 – 37.4)	37.3 (36.8 – 37.4)	

Data were expressed as mean ± SD or median (range). * p < 0.05, ** p < 0.01, *** p < 0.001, before versus during KC and † p < 0.05, † † p < 0.01, during versus after KC. CV: coefficient of variance, rSO_2_: regional cerebral oxygenation.

### Power spectral analysis

**Figure 2:**
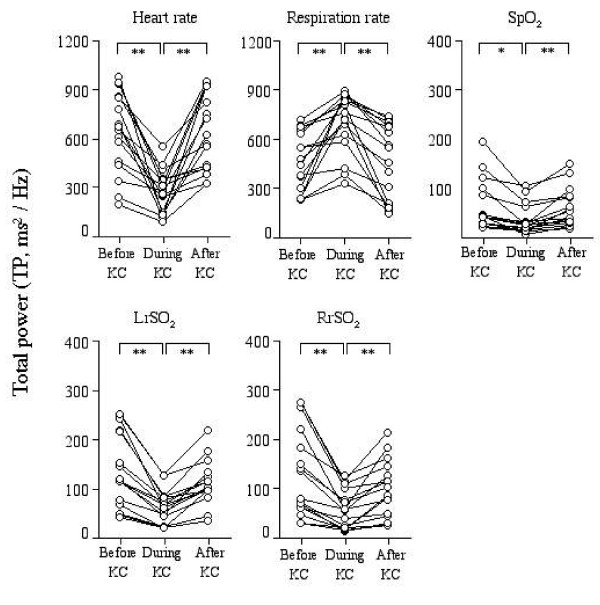
**Individual values of total power (TP) of power spectral density before, during, and after KC, displaying visually qualitative changes during KC**. A repeated measures ANOVA was performed to determine the statistical differences among the three sessions: before, during, and after KC. * p < 0.01, ** p < 0.001, before vs during KC or during vs after KC.

Changes in TP during the sessions were shown in Figure 2. The TP of HR was significantly decreased during KC and increased after KC (before: 635 ± 280, during: 268 ± 134, after: 618 ± 240 ms^2^/Hz; before vs during and during vs after, p < 0.01). The TP of SpO_2 _had a similar tendency to that of HR. On the other hand, the TP of RR was significantly increased during KC and decreased after KC (before: 458 ± 186, during: 713 ± 175, after: 461 ± 231 ms^2^/Hz; before vs during and during vs after, p < 0.01). In regional cerebral oxygenation, the TP of either LrSO_2 _or RrSO_2 _was significantly decreased during KC and increased again after KC (LrSO_2_: before: 121 ± 91, during: 54 ± 41, after: 90 ± 56 ms^2^/Hz; RrSO_2_: before: 123 ± 69, during: 55 ± 30, after: 98 ± 46 ms^2^/Hz; before vs during and during vs after, p < 0.001, respectively in each parameter).

**Figure 3:**
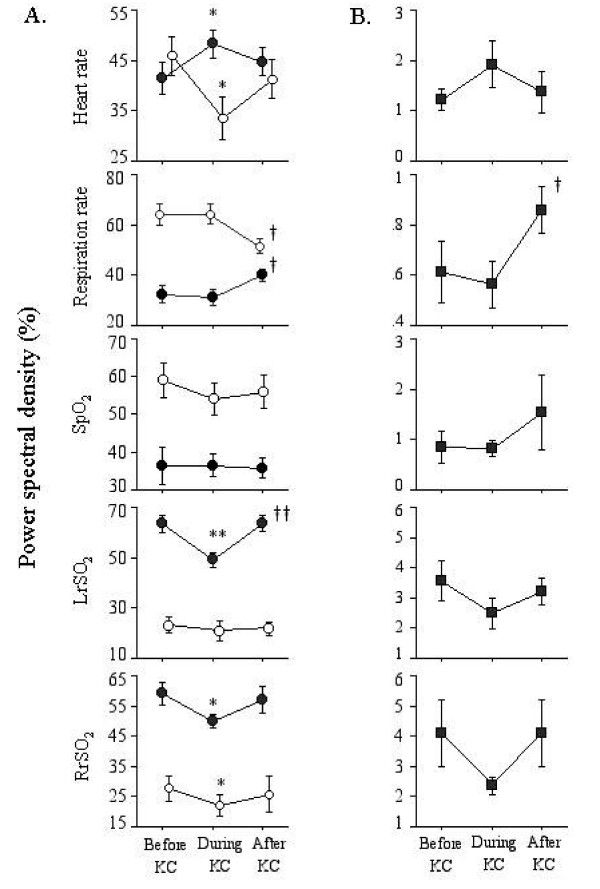
**A: Power spectral density in low – frequency (LF: close circle) and high – frequency (HF: open circle) bands before, during, and after KC.** LF and HF are expressed as normalized values (%LF = LF/total power × 100 and %HF = HF/total power × 100). B. The ratio of LF/HF before, during, and after KC. Data is presented as means ± SEM. A repeated measures ANOVA was performed to determine the statistical differences among the three sessions: before, during, and after KC. * p < 0.05, ** p < 0.01, before vs during KC. † p < 0.05, † † p < 0.01, during vs after KC.

The percentages of LF, HF, and the ratio of LF/HF are shown in Figure 3A and 3B. For HR, the %LF was significantly increased during KC (before: 41.4 ± 11.5, during: 48.3 ± 9.8%, p < 0.05), while %HF was decreased (before: 45.9 ± 13.9, during: 33.5 ± 15.3%). In contrast, the %LF of rSO_2 _was significantly decreased during KC for both LrSO_2 _(before: 63.5 ± 11.2, during: 49.2 ± 9.2, after: 63.8 ± 10.2%; before vs during and during vs after, p < 0.01) and RrSO_2 _(before: 59.1 ± 11.5 during: 49.9 ± 6.9, after: 57.0 ± 13.6%; before vs during, p < 0.05). The %HF was decreased during KC for RrSO_2 _(before: 28.1 ± 13.8, during: 22.6 ± 10.9, after: 26.2 ± 19.2%; before vs during, p < 0.01), although there was no significant change in this parameter for LrSO_2 _during the sessions. For RR and SpO_2_, significant changes were not observed in %LF or HF during KC, although the %LF of RR after KC was significantly increased while the %HF was decreased (p < 0.05, respectively) with an increased LF/HF ratio (p < 0.01). There were no significant differences in the ratio of HR, SpO_2_, LrSO_2_, and RrSO_2 _during the sessions (Figure 3B).

At the day of KC, four infants had received theophylline. Infants who received theophylline showed the similar tendency to those without theophylline during KC and after KC.

### Behavioral analysis

**Figure 4:**
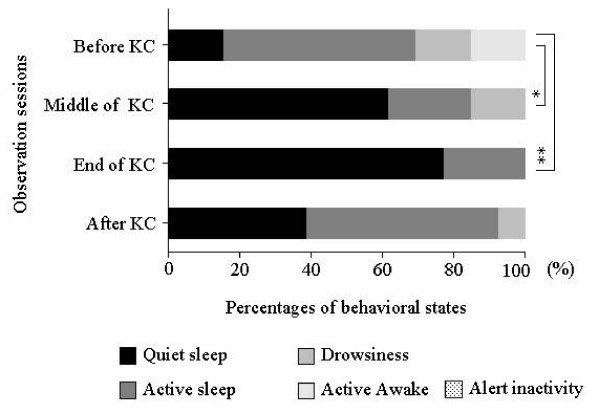
**Behavioral states of infants observed before KC, in the middle of KC, at the end of KC, and 30 minutes after KC.** The quiet sleep state was remarkably increased in the middle of KC and at the end of KC. A chi square test was performed to determine the significant differences in the four behavioral states.* p < 0.05, ** p < 0.01, before versus middle of KC or before versus end of KC.

The behavioral states during the observation period are shown in Figure 4. The percentage of infants with quiet sleep states remarkably increased during KC (61.5%) compared to those before KC (15.4%). This percentage increased further to 76.9% at the end of KC and decreased to 38.5% at 30 minutes after KC. In contrast, the percentage of infants in active sleep was 53.8% before KC, decreased to 23.1% in the middle of KC, and increased to 54.0% at 30 minutes after KC.

## Discussion

In this study, regional cerebral oxygenation using NIRS was measured during KC in addition to cardiorespiratory parameters in stable low birth weight infants and analyzed by using power spectral analysis. By conventional analysis, the CV of HR was decreased, and mean RR and body temperature were increased during KC as previously reported [7,9,14]; however, significant changes were not found in mean rSO_2_. By power spectral analysis, TP was decreased in rSO_2_; HR, and SpO_2 _during KC, whereas the TP of RR was increased. Further, the LF of rSO_2 _was found to be decreased during KC while the LF of HR was increased with decreased HF. This study has shown that the spectral characteristics of cerebral oxygenation are significantly different between during KC and before KC or after KC as well as HR, RR and SpO_2_; however, there were no significance differences in their mean values. The power spectral analysis was found as a more effective analytic approach for revealing the physiological responses compared to the conventional analysis.

In spectral analysis of heart rate variability (HRV), low – frequencies (0.02 – 0.20 Hz) reflect sympathetic activities such as the baroreceptor reflex, high-frequencies (0.2 – 2.0 Hz) reflect parasympathetic activities such as vagal activity, and the ratio of LF/HF power reflects the balance between sympathetic and parasympathetic activities [23-26,32]. Several studies have been published on autonomic function of HRV in preterm infants and only two studies had been previously reported on HRV during KC intervention using power spectral analysis up to now. One of them showed a decrease of LF and HF during KC [27], while the other showed an increase of LF during KC [28]. In our study, the LF of HR was increased during KC and the LF of rSO_2 _was decreased. In general, the increase in sympathetic activities and decrease in cerebral oxygen delivery with the head in an up-tilting position are assumed to be due to gravity causing pooling of blood and thereby activating baroreceptors as previously reported [29,33], and the decrease of LF of rSO_2 _during KC with increased LF of HR described in this study might be supported previous studies. These results could be understood as activation of the central nervous system and brain function during KC position. Besides these, the increase in body temperature and respiration observed in our study may have an effect on the LF of HR as previously reported on HRV [28,34]. Further, TP is the index of total variance in power spectral analysis and changes in TP during KC indicate the changes in total variance. In our study, TP of rSO_2_, HR, or SpO_2 _have been shown to decrease during KC. A possible explanation for these decreases during KC might be associated with reduced activity and increased quiet sleep state during KC as previously reported [35,36]. These results could be interpreted as physiological stability elicited by KC intervention. Although the dominance of HF has been reported in HR during the quiet sleep state [37,38], this was not observed during KC in a previous study [28] or in the current study. The position of the head of infants during KC might be responsible for the discrepancy between the left and right rSO_2_.

To our knowledge, the present study was the first study to analyze the response of rSO_2 _due to KC in LBW preterm infants through spectral characteristics. Therefore, it was difficult to compare the results of this study with other similar studies and apply to the clinical settings. Despite the sampling limitation, this reference data provide new understanding into the response of cerebral hemodynamics in preterm infants and should have significant implications to generalize the rSO_2 _responses with different modalities in LBW infants. Further studies are necessary to determine the clinical relevance of the present findings.

## Conclusion

The results of this study revealed that changes of cerebral hemodynamics associated with KC position in preterm infants as well as cardiorespiratory parameters. These changes were especially apparent by power spectral analysis. Furthermore, the results of this study indicate that KC may contribute to the activation of central nervous system and brain function. Further study is needed to determine the underlying physiology responsible for these differences.

## Abbreviations

KC: Kangaroo care; LBW: low birth weight; NICU: neonatal intensive care unit; FFT: fast Fourier transformation; LrSO_2_: left side regional cerebral oxygenation; RrSO_2_: right side regional cerebral oxygenation; HR: heart rate, RR: respiration rate; TP: total power; HF: high frequency; LF: low frequency; weeks, wks; NIRS: near infrared spectroscopy, ECG: electrocardiogram, HRV, heart rate variability.

## Competing interests

The authors declare that they have no competing interests.

## Authors' contributions

EAB: Participated in all data collection, in the analysis and discussion of the results, and in the writing of the manuscript; MB: Mainly worked with first author and responsible for this study.; NO: Participated in all clinical and physiological data collection; SY: Participated in organized the study group with nursing staff.; ST: Participated in interpretation of clinical findings and theoretical support; HY: Introduced and established the NICU local area network system (LAN system) for physiological data recording and Participated in clinical suggestion about physiological rhythmicity; MK: Participated in theoretical support of physiological instability in neonates and physiological rhythmicity; YK: Organized the study group and checked the manuscript finally.

## Pre-publication history

The pre-publication history for this paper can be accessed here:


